# Trinity of G-tetrads and origin of translation

**DOI:** 10.1186/s13062-022-00327-9

**Published:** 2022-05-31

**Authors:** Besik Kankia

**Affiliations:** 1 Department of Chemistry and Biochemistry, The Ohio State University, Columbus, OH 43210 USA; 2 Institute of Biophysics, Ilia State University, 0162 Tbilisi, Republic of Georgia

**Keywords:** Quadruplex world, RNA world, G-tetrad, Proto-tRNA, Origin of translation, Prebiotic, Genetic code

## Abstract

**Background:**

The RNA world hypothesis cannot address most of the questions of the origin of life without violating the continuity principle (small Darwinian steps without foresight and miracles). Moreover, the RNA world is an isolated system incapable of accommodating the genetic code and evolving into extant biochemistry. All these problems are rooted in the central assumption of the hypothesis: de novo appearance of the ribozymes, production of which represents a multistep reaction requiring the complementarity principle. Thus, even the basis of the RNA world is at odds with the continuity principle—it uses foresight (multistep reaction) and a miracle (complementarity principle). Can a three-dimensional (3D) architecture, capable of molecular recognition and catalysis, be formed in a single-step reaction without the complementarity or any other preexisting rules?

**Hypothesis:**

At first glance, the above question sounds rhetoric since the complementarity principle is the essential feature of the RNA world; it turns an RNA polymer into a genetic material. Without it, the RNA world becomes as shapeless and unconvincing as other hypotheses based on the non-hereditary molecules (i.e., protein world). However, it was suggested recently that the quadruplexes could initiate life and take necessary evolutionary steps before the arrival of the complementarity rules. The hypothesis relies on the unique properties of guanines (Gs) to self-assemble into G-tetrads and efficiently polymerize without any external help or preexisting rules. Interestingly, polyG folds into an unusually stable and well-structured monomolecular architecture that uses the quadruplex domain (QD) assembly. The QD has a strictly defined zigzag-like building pattern to accommodate only three G-tetrads. Since both QD architecture and codon length are based on triplets, the inevitable question arises: are they related? Or could QD play the role of the early adapter and determine the codon length? The current paper is an attempt to answer this question.

**Conclusion:**

While without translation apparatus most of the steps of the extant translation are physically impossible, the QD-mediated translation is sterically feasible and can be explained by physicochemical properties of the QD and the amino acids without violating the continuity principle. Astonishingly, the quadruplex world hypothesis can address all the shortcomings of the RNA world, including its most significant challenge—step-by-step evolution from the polymerization of the first polynucleotide to the extant biochemistry.

## Background

### Genotype–phenotype relationship in the RNA world

**Fig. 1:**
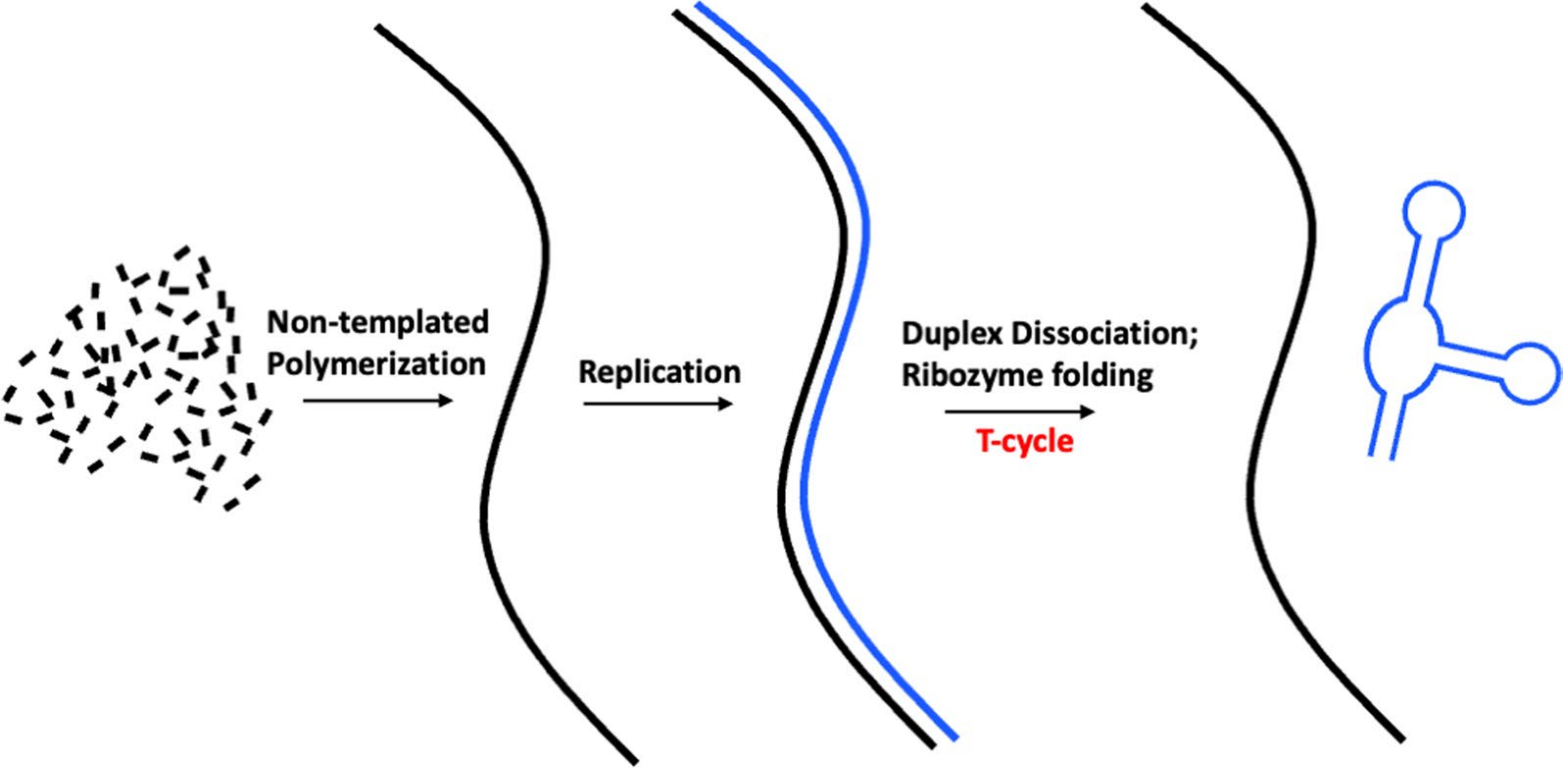
Emergence of ribozymes in the RNA world. The RNA template (black line) is polymerized form free nucleotides (black segments). The replicons (blue) fold into a ribozyme upon the heating/cooling cycle (T-cycle). Structural reorganization of the template is omitted for simplicity

Despite the inability to address virtually any significant questions of the origin of life, the RNA world hypothesis is the only theory that avoids the chicken-or-egg conundrum. RNA is capable of both (i) encoding the genetic information; and (ii) folding into a three-dimensional (3D) architecture responsible for molecular recognition and catalysis. Thus, an RNA molecule can represent both the genotype (information) and the phenotype (3D architecture responsible for the catalysis). In the extant biochemistry, the genotype–phenotype relationship is governed by the translation apparatus (the ribosome and associated factors). The information encoded in a gene is accurately translated into a polypeptide and folded into a functional protein by the same apparatus. As a result, the process is highly programmable—the information encoded in the genotype is translated into the phenotype with high accuracy and reproducibility. In contrast, in the RNA world, the genotype–phenotype relationship is rather vague since the simple base-pairing thermodynamics determines it. Usually, an RNA molecule adopts a 3D structure with the lowest folding free energy. However, this is not a straightforward process due to the problems in the RNA folding: (i) existence of more than one structure with similar free energies, (ii) stable mismatches (e.g., G•U), or (iii) kinetically trapped misfolded intermediate structures. Thus, the genotype–phenotype relationship in the RNA world is not an unambiguous process. Furthermore, the genotype production consists of two problematic steps, non-template polymerization of the very first strand (antisense template) and the template-directed replication (Fig. 1). The former is inhibited by hydrolyses [1, 2] and cyclization [3–6], while the latter is very inefficient and characterized by low fidelity [7]. Thus, the non-enzymatic production of a ribozyme represents the sequence of three problematic reactions unable to overcome the Eigen threshold and initiate the evolution of the extant translation apparatus [8]. Additionally, since the ribozyme production mechanism represents a combination of three conceptually different processes (Fig. 1), it violates the continuity principle (no foresight evolution). Thus, even avoiding the chicken-or-egg conundrum is ostensible in the RNA world hypothesis.

### Triplet nature of the genetic code

One of the most mysterious features of the genetic code is the triplet nature of the coding mechanism, which could be explained by the fact that the triplet code represents the smallest combination of the 4-letter alphabet (G, C, A, U) that could encode all 20 amino acids (4^3^ = 64 permutations). The genetic code might start as a singlet system with the capability to code only four amino acids (4^1^ = 4), expanding to the duplet code with 16 permutations (4^2^ = 16), and then finally reaching the needed coding capacity. Indeed, the universal genetic code’s apparent features suggest the code expansion. The most noticeable one is the degeneracy/redundancy of the code. Eight amino acids (Gly, Ala, Pro, Arg, Ser, Val, Leu and Thr) are coded by codons with fourfold degeneracy in the third position (so-called meaningless positions). Interestingly, almost all amino acids are the simplest, and the most frequent nucleotides in the meaningful positions, are G and C bases. These features can be explained by initiating the code as a 1-letter (G) or 2-letter (G and C) alphabets coding the simplest amino acids and gradually expanding to the extant system [9–11]. The easiest way to expand the code would be codon elongation. However, the codon length alteration would lead to mistranslation of proteins and thus a complete loss of evolutionary fitness. Therefore, the genetic code must have been a triplet code from the very beginning [12]. Also, if the driving force behind the selection of the triplet code was to encode all 20 amino acids, why did the evolution not select more optimal codes? For instance, the 6-letter duplet (6^2^ = 36) or the 2-letter quintuplet (2^5^ = 32) systems can encode 20 amino acids with ~ 60% efficiency while the extant code's efficiency is only 30%. It seems that the triplet nature of the genetic code is not a result of natural selection. It is instead determined by stereochemistry between the triplet and an early adaptor, or proto-tRNA [12]. However, the tRNA or any other molecule employing base-pairing as a structural element could not play a role in the proto-tRNA since the diameter of the double-helical RNA, 2.3 nm, is significantly larger than the length of the triplet, ~ 1 nm. Alternatively, a direct stereochemical relationship between the amino acids and the triplets could be suggested as a reason for the code’s triplet nature. However, the amino acid contour length, ~ 0.35 nm, is significantly shorter than the triplet. Thus, neither RNA duplex nor amino acids can determine the codon length.

### The de novo appearance of the replication process is doubtful

Above listed problems or any other issues of the RNA world, are different tips of the same iceberg—the primary problem is hidden in the properties of the genetic code. The evolution of the code is driven by minimization of the translation errors, which means that the extant translation apparatus must be the result of the coevolution of all the components [13–15]. The problem is so complex and obscure that considered practically unsolvable [16].

Since all attempts to address the various issues of the RNA world are eventually failing, there must be something fundamentally wrong with the hypothesis. It was suggested that the problem could be caused by de novo appearance of the replication process [17]. A premature launch of the replication not only affects chronology of the evolution but isolates/disconnects the anachronic step from the entire process.

To restore the natural flow of the evolution, we need to find a nucleic acid precursor of RNA, which in the absence of the complementarity rules (i) emerges and self-reproduces; (ii) establishes the unambiguous genotype–phenotype relationship, (iii) determines the triplet nature of the genetic code; (iv) allows coevolution of the genetic code and the translation; and (v) allows step-by-step evolution of the complementarity principle and the replication. Thus, the precursor must take several necessary steps before launching the replication. Since complementarity is the essential and only principle of genetics, at first glance, finding such a molecule seems unrealistic. However, a closer look at the nucleic acid quadruplexes reveals that this is feasible [17].

## Quadruplex world hypothesis

**Fig. 2:**
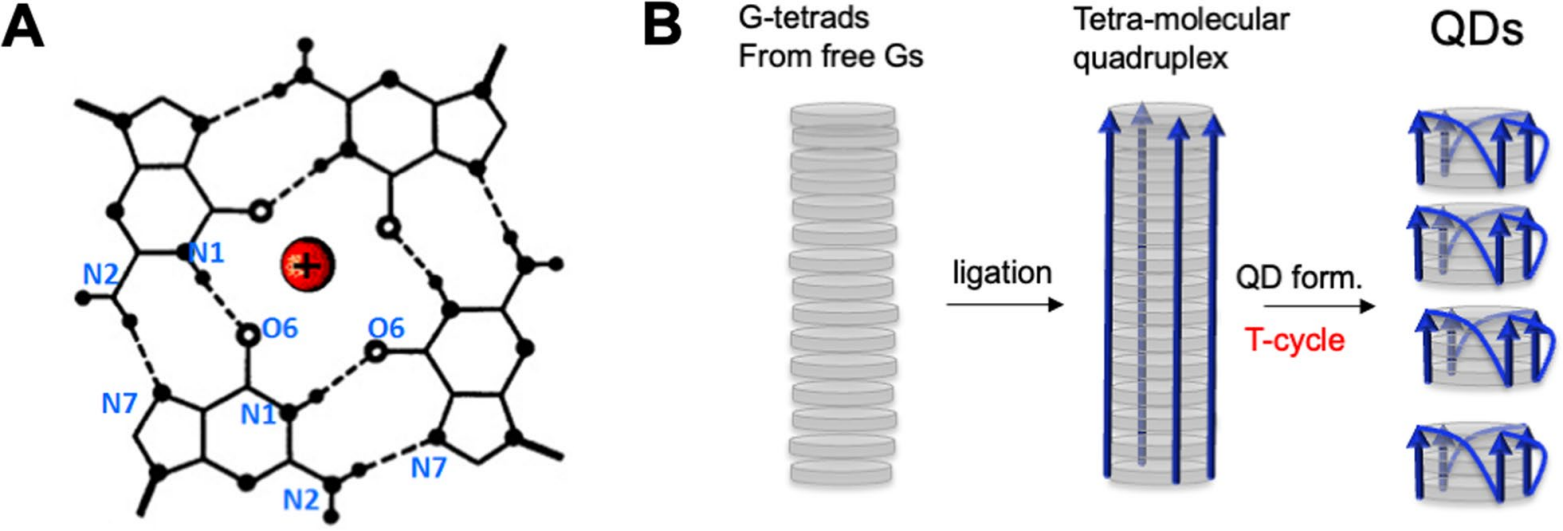
Emergence of the quadruplex domain (QD) in the quadruplex world. **A** G-tetrad with cation in the center. **B** Steps in prebiotic polymerization of quadruplexes from free guanines (G) stacked into G-tetrads. Only G-tetrads (gray disks) and phosphodiester backbone (arrows) are shown. Curved lines in QDs correspond to Z-shaped G-loops

**Fig. 3:**
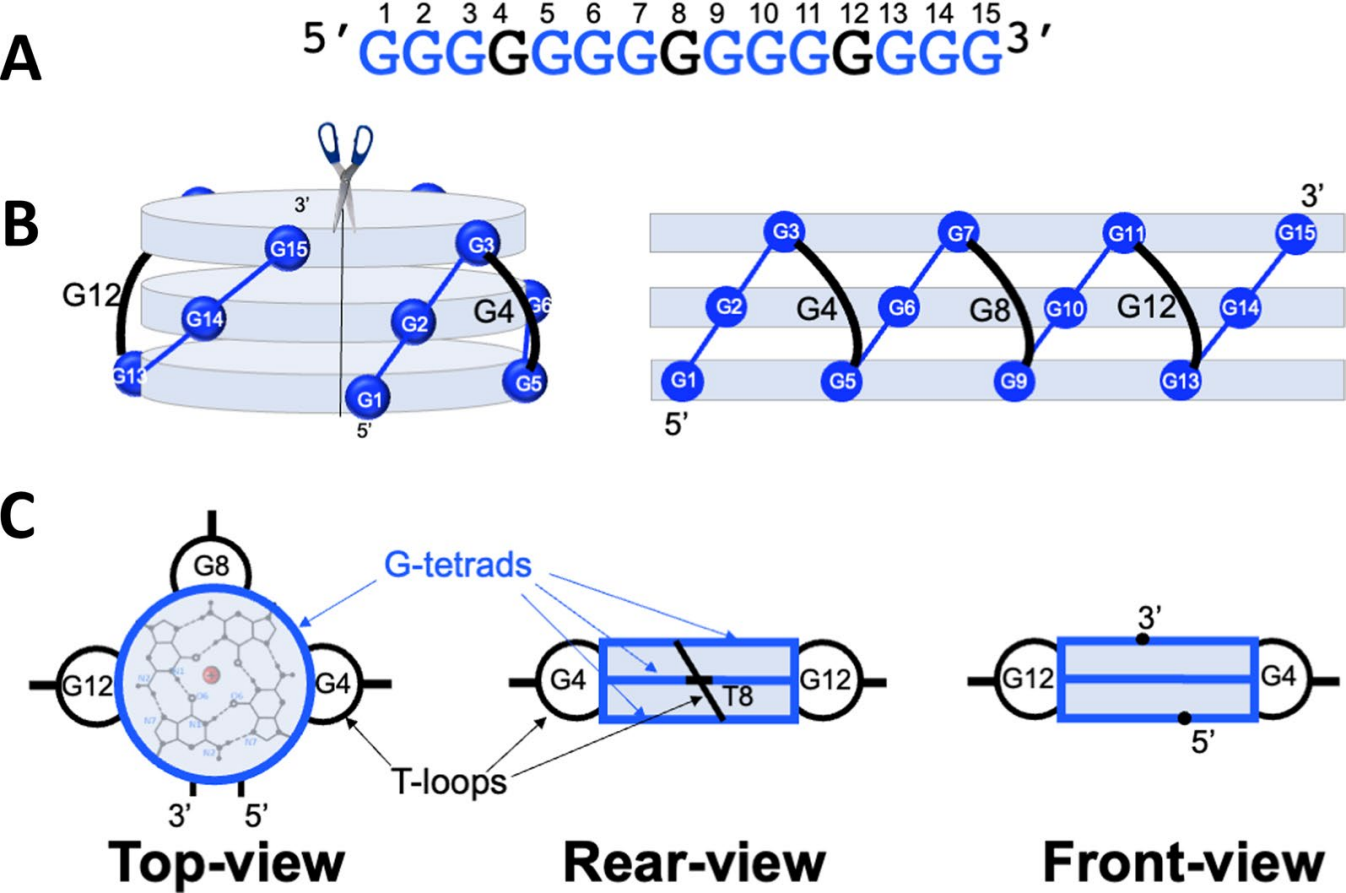
QD domain. **A** G_15_ oligomer using G4, G8 and G12 (black) as Z-loops to form QD architecture. The Gs involved in G-tetrads are shown in blue. **B** 3D representation of the QD and surface of the model unwrapped in 2D map. Grey discs represent G-tetrads and black curved lines correspond to G-loops. **C**: Schematic representation of the QD from the different views

The quadruplex world can address most of the deficiencies of the RNA world hypothesis without violating the continuity principle. For instance, it suggests reasonable explanations for (i) the polymerization of the very first nucleic acid; (ii) the origin of homochirality; and (iii) RNA-to-DNA transformation [17]. The hypothesis is based on the ability of free guanines (Gs) to assemble into square-planar metal complexes or G-tetrads (Fig. 2). The G-tetrads form highly organized crystalline structures with a similar helical parameter as quadruplexes made of polyG strands. As a result, the reactive moieties of the free Gs are juxtaposed facilitating the polymerization/condensation. Thus, in the quadruplex world, life originates directly from the organic G-crystals capable of producing a standard nucleic acid strand, polyG, without any external help (i.e., clay minerals [3, 4, 18]. After reaching the 15-nucleotide (nt) length, G_15_, the strands form monomolecular quadruplexes with strictly defined 3D architecture [17]. The structure, hereafter called quadruplex domain (QD) (Fig. 3), consists of four parallelly aligned GGG-segments connected through three Z-shaped loops (they are also known as propeller or double chain-reversal loops) [19]. Figure 3 demonstrates QD with the single-nt G-loops, but loops can be made of other nucleotides, abasic sites or oligomers (for more details see section "A single nucleotide can form Z-loop").

The genotype production and folding processes are significantly straightforward and more accurate in the quadruplex world. Specifically, the genotype production is eventually a single-step process—self-assembly of free Gs accompanied by the polymerization of four identical strands (Fig. 2). Thus, the quadruplexes are truly self-assembling and self-replicating molecules. In contrast, the genotype production in the RNA world is the two-step reaction: (i) non-template polymerization of the precursor (or antisense strand), which relies on the external help [18, 20, 21]; and (ii) precursor-directed polymerization of the replicons based on the complementarity principle. Thus, the genotype production in the RNA world is a complex process requiring the external help, the complementarity principle and involves two different molecules, the antisense and sense strands.

The phenotype folding in the quadruplex world is very straightforward as well. The QDs with the single-nt loops are the most stable known structural motif in the entire nucleic acid world [22, 23]. The exceptional stability of QDs, attributed to the all-parallel alignment of GGG-tracts, centrally located cations and the Z-shaped loops, ensures that G_15_ or longer oligomers fold only into the QD architecture (Fig. 3). The QD formation is characterized by fast kinetics allowing the folding even upon a rapid cooling on ice, uncharacteristic to RNA structures requiring careful annealing to avoid misfolded structures. Thus, the genotype–phenotype relationship in the quadruplex world is a highly programmable and unambiguous process.

**Fig. 4:**
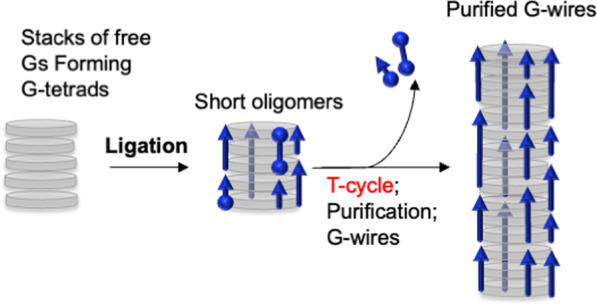
Purification of oligoGs upon the heating/cooling cycles (T-cycles). Separation of oligoGs (blue arrows) from contaminated oligos (shown as blue segments with spheres) and assembly of pure G-wires. The gray disks correspond to G-tetrads

The temperature cycles are considered the most prominent energy source in abiogenesis [24]. Production of both RNA and quadruplexes (Figs. 1 and 2) involves dissociation/unfolding of the multimolecular assemblies and forming the final monomolecular phenotypes. In the RNA world, the unfolding steps can also have adverse effects if the polymerization sessions are longer than the temperature cycle. In this case, incomplete/nonfunctional RNA will be produced. In contrast, the temperature cycles fit perfectly into the quadruplex world and create additional opportunities. For instance, short oligoGs usually slip against each other and form multimolecular G-wires [25–27] (Fig. 4). This feature could be beneficial during abiogenesis when combined with the temperature cycles. For instance, it could serve to purify quadruplexes from impurities such as other nucleotides or incorrect G-enantiomers (Fig. 4) [17].

In conclusion, three critical features should be mentioned in the quadruplex world: (i) production of the oligoG is a single-step, self-assembling, and self-replicating process; (ii) the process is very efficient with purification/enriching mechanism eliminating the contamination; (iii) G_15_ or longer oligomers folds into the QD with almost 100% programmability; in other words, the genotype–phenotype relationship is strictly unambiguous. Thus, the quadruplex world represents an error-free "one-trick pony" but is ready to expand its repertoire upon adding the complementarity rules.

## Unique properties and primordial production of QD

**Fig. 5:**
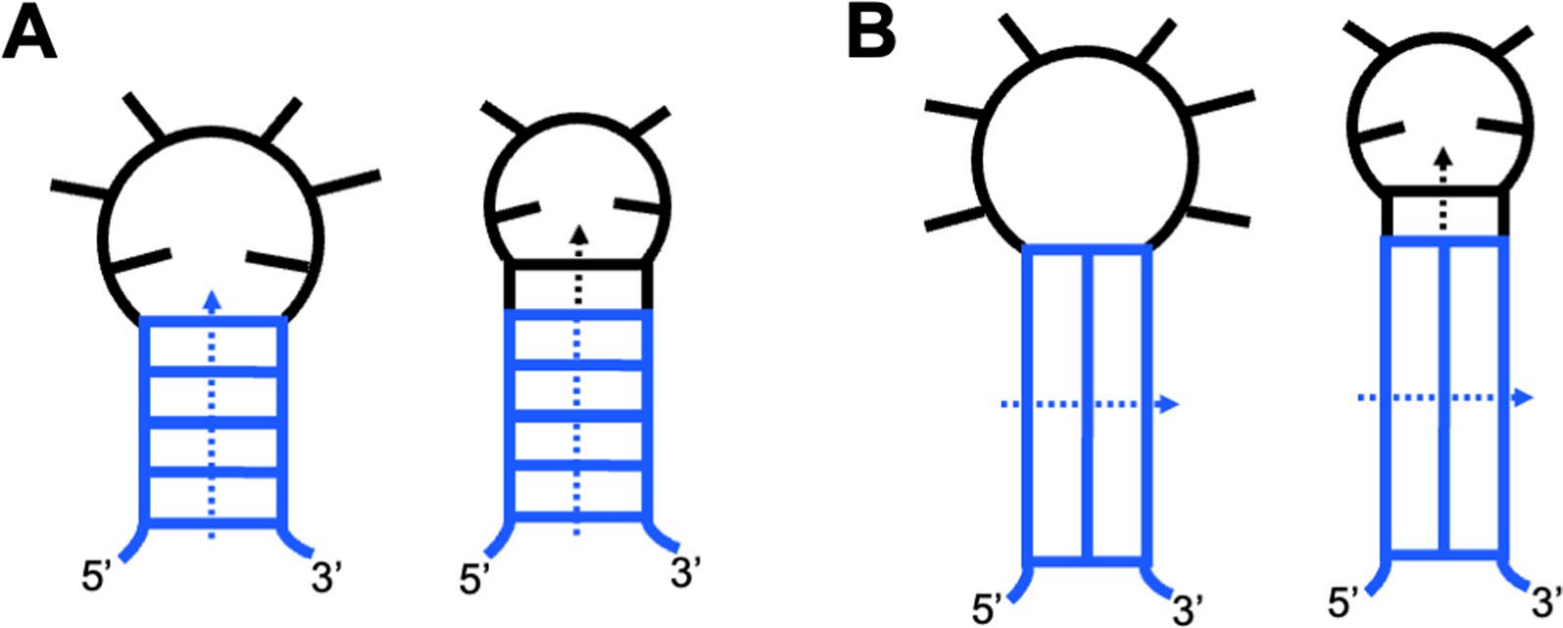
Diagrams of base-pairing in U-shape (**A**) and Z-shaped (**B**) loops. Parental structures (duplex in panel A and quadruplex in panel **B**) are shown in blue; loops and base-pairs in the loops are shown in black

Nucleic acids form intramolecular structures by looping of two or more segments of a strand. The loops are typically unstructured portions of the same strand, linking the interacting segments and turning entropically unfavorable intermolecular interactions into favorable intramolecular structures. The linkages can be grouped according to the strand orientation of the resulted structures: (i) U-shaped loops between antiparallel segments (Fig. 5A) and (ii) Z-shaped loops between the parallel segments (Fig. 5B). U-loops usually link nucleotides of the same structural element (base pair, base triad, or G-tetrad), while Z-loops, documented only for quadruplexes, link Gs of different G-tetrads. Because of the Z-loops, the quadruplexes can fold into all-parallel topology with strictly defined and unique structural properties. Many unusual properties of the QD are discussed elsewhere [17]. Here, several specific features of Z-loops, essential to the understanding role of QDs in the primitive translation, are discussed.

### Z-loops accommodate only three G-tetrads

It was shown earlier that the all-parallel quadruplex with three Z-loops could accommodate only three G-tetrads [28]. Specifically, single-nt loops ideally stretch over three G-tetrads and form the most favorable known structure among whole the nucleic acid world. It seems reasonable to assume that by simultaneous elongation of the G-tracts and the loops one can increase the number of G-tetrads and form longer QDs. However, the architecture, shown in Fig. 3, can accommodate only three G-tetrads. For instance, (T_2_G_4_)_4_ folds into an anti-parallel quadruplex with two U-shaped GTTG and TTG loops and one Z-shaped TT loop [29]. Moreover, when a quadruplex is forced to adopt the all-parallel topology by employing an all-RNA sequence (RNA is not able to adopt syn glycosyl bonds required for U-loops) it still is not able to accommodate more than three G-tetrads—RNA sequence (G_4_C_2_)_4_ assembles only three G-tetrads connected by three extra-long Z-shaped GCC loops [30]. Thus, the triplet nature of QD is strictly defined.

### Orientation of Z-loop bases and arms: QD as an embryo of tRNA

**Fig. 6:**
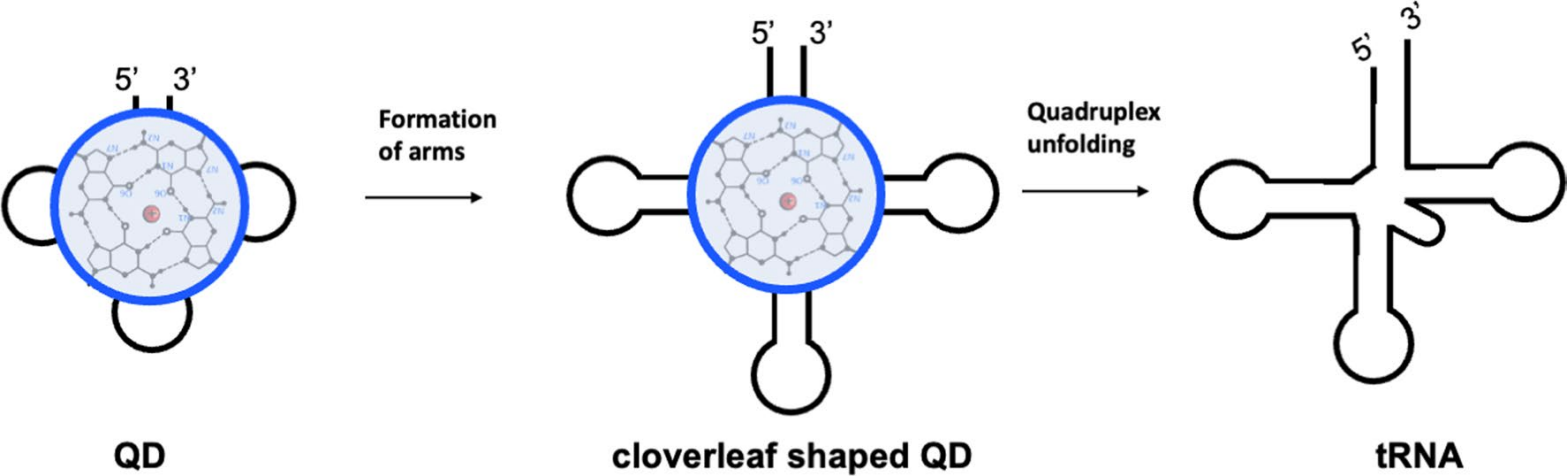
A scenario of QD transformation into tRNA. The clover shaped QD can be formed by the loop elongation and stem addition. The quadruplex unfolding finalizes the process by forming a standard four-way junction

Z-shaped loops are stretched over quadruplex grooves without crossing the helical axes (Fig. 5B). In contrast, U-shaped loops circle the loop-closing base pair and cross the helical axes (Fig. 5A). This predisposes U-loops’ bases to stack to the loop-closing base pair, while bases of the Z-loops are exposed almost perpendicularly to G-tetrads and are better available for intramolecular pairing [31]. Because of the same reasons, U-shaped loops can extend or shorten the stem part of the hairpin upon mutations (nucleotide substitution, insertion, or deletion). However, the mutations in Z-loops can create double-helical arms perpendicular to the parental G-tetrad stack without affecting the length of the G-tetrad stack (Fig. 5). This not only keeps terminal G-tetrads accessible for stacking to each other and multimerization [31, 32] but allows a straightforward transformation of QD into a tRNA (Fig. 6). Specifically, QD is arguably the shortest and the simplest nucleic acid construct that folds into the stable and complex architecture. The stack of three G-tetrads represents a platform with evenly distributed locations to grow the arms. The terminal Gs are in close vicinity and upon extending can form a double-helical stem. Similarly, elongation of the loops can create stem-loop structures. Thus, the stack of G-tetrads resembles a "quadruplex knot", which upon a simple modification (single-nt substitution, deletion, or insertion) or loosing of the chelated cation can unfold and transform into a standard four-way junction. At this point, QD is progressed into a cloverleaf-like structure similar to tRNA (Fig. 6). This model of tRNA origin considers the formation of the cloverleaf shape from a monomolecular structure, which is entropically favorable; all other models consider dimerization or fusion of separate minihelices [16, 33, 34].

### A single nucleotide can form Z-loop

As discussed above, single-nt loops demonstrate the highest thermodynamic stability of QDs, for both RNA and DNA analogs [35]. The most favorable is the abasic sites [36]. Attaching of the pyrimidine bases induce slight destabilization (~ 1 °C per base) and purines further destabilize the structure (~ 8 °C per base) [36, 37]. This should be attributed to the stronger stacking interactions of the purines with adjacent guanines in unstructured QD, which must be overcome during rearrangement of the sequence into the quadruplex. Elongation of the quadruplex loops is usually accompanied by destabilization of the structure [36, 38–40].

While QDs with the single-nt loops demonstrate the same all-parallel architecture for DNA and RNA sequences, loop elongation has different effects. Specifically, the loop modifications cannot affect the all-parallel topology in RNA quadruplexes. The structural monomorphism of the RNA sequences is attributed to RNA's incapability to adopt syn glycosyl bonds required for U-looping of the antiparallel strands. In contrast, the loop elongation in DNA quadruplexes is accompanied by the formation of antiparallel topologies through U-looping. However, under specific experimental conditions (i.e., low water activity) DNA domains with even three-nt loops maintain their all-parallel topology [41].

Thus, the QDs, can be formed with single-nt loops and can be elongated without structural changes, especially for RNA-QD. This is important for the genetic code expansion from the singlet to triplet codons (see section “QD as a proto-tRNA” below). In contrast, double-helical RNA stem-loops require at least four nucleotides, which is already longer than the codon length and demonstrate the incapability of tRNA or any other RNA-based adaptors to accommodate a similar genetic code expansion mechanism.

### Non-enzymatic production of QDs

The QD constructs could be produced in several different ways: (i) the template-free synthesis from Gs (Fig. 2B) [17]; (ii) the quadruplex-templated and catalyzed ligation of different strands [42]; and (iii) the template-directed replication using the complementarity principle (Fig. 7 in [17]). Thus, the first two methods rely on the G-tetrads as the structural and recognition element and could produce QDs before establishing the complementarity principle.

**Fig. 7:**
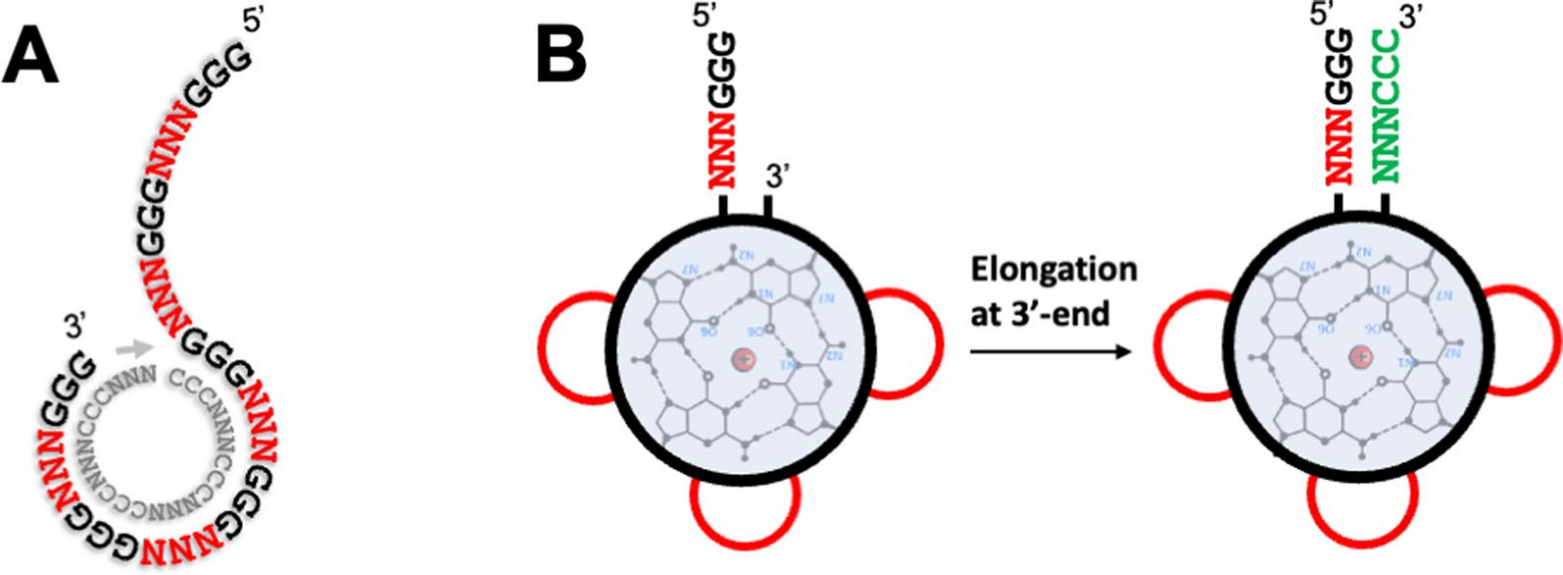
Replication-based production of QDs with the operational code. **A** The rolling circle replication (RCR) of QDs with three-nt loops—(G_3_N_3_)_4_G_3_. **B** QD with the operational code, NNN, in the stem. The red curves correspond to NNN loops and green segment is the sequence replicated after the QD folding

*The template-free production* is based on the capability of free Gs to (i) form G-tetrads stacked on each other with similar helical parameters as quadruplexes [43–45] and (ii) self-polymerize with canonical 5'-3' phosphodiester backbone non-enzymatically (Fig. 2B) [46–48]. After reaching the required length, G_15_, oligomers fold into monomolecular QDs. This method is the simplest and represents a controlled self-polymerization process directly from G-monomers without any preexisting rules or external help. The formation of the single-G loops is suggested from the thermodynamic properties of QDs. Specifically, single-nt loops are thermodynamically more favorable than longer loops [36, 38–40]. The C or U nucleotides could have been incorporated at loop positions after spontaneous depurination of Gs not involved in G-tetrads (see Fig. 7B in [17]).

*The quadruplex-templated and catalyzed ligation* is also based on tmDNA (tetrahelical monomolecular DNA) and QD architectures and capability of the truncated quadruplexes, so-called quadruplex couplers, to play the role of a catalyst [42]. It facilitates the ligation by juxtaposing two QD constructs’ reactive moieties and demonstrates multiple turnover activity.

*The template-directed replication* is based on the complementarity rules and could have produced QD constructs through rolling circle replication (RCR) (Fig. 7B in [17]). This method could be convenient in producing QDs with longer loops through imperfect priming or strand relocation/slippage. This method can produce long tmDNA polymers (several QDs covalently attached to each other), but the formation of single QDs could have been favored by spontaneous dissociation of the quadruplexes upon replication. This feature was successfully used earlier to design self-dissociative primers for isothermal PCR [49]. Additionally, RCR can include the operational RNA code for amino acids [50–52] in the stem when replicon contains more than four repetitive units (Fig. 7).

## QD as a proto-tRNA

As discussed above, one of the weakest points of the RNA world hypothesis is its failure to explain the inseparable nature of the genetic code evolution and the translation process. The translational apparatus consists of several physical components: tRNA, aminoacyl-tRNA synthetase, ribosomes. While all of them are essential, tRNA is the crucial molecule; one can imagine translation without aminoacyl-tRNA synthetase or ribosomes but not without the mediator between nucleic acids and proteins. So, the most obvious way to explain the inseparable nature of the genetic code and the translation process would be to find a multifunctional precursor of tRNA (proto-tRNA), which alone could conduct a primitive translation and step-by-step evolve into the extant translation apparatus. Since both QD architecture and codon length have the exact triplet nature, here arises the question: is it possible that the QD played the role of proto-tRNA and determined the codon length? Before analyzing this possibility, the requirements for a suitable proto-tRNA are listed.

**Fig. 8:**
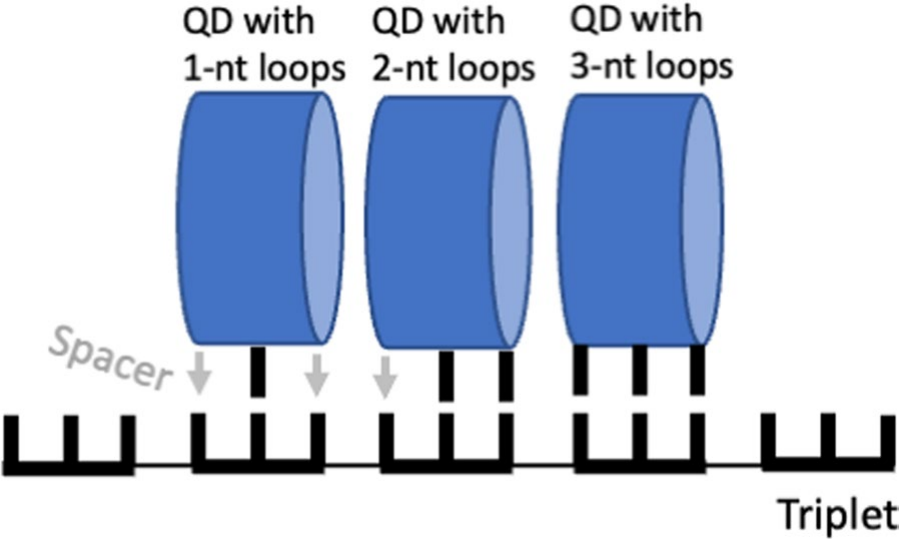
Triplet offset mechanism in QD. The QDs are represented as cylinders and the black bars correspond to the loop bases involved in base-pairing (meaningful positions). The meaningless positions are shown by gray arrows, or spacers

*First*, an appropriate adaptor had to be a well-structured molecule with a distinctive/invariable geometric feature matching the length of the codon. This is important since two or more adaptors should bind simultaneously to adjacent codons on the same mRNA without skipping a nucleotide between them. In other words, the adaptor should have a distinctive "measuring stick" that closely matches the codon length (see Fig. 8).

*Second*, to expand the code without the codon length elongation, the adaptor had to be able to create two kinds of codon positions: the meaningful positions involved in the direct binding (i.e., base pairing) between the triplet and the adaptor and the meaningless positions, or "spacers" not involved in the binding (Fig. 8). In other words, the adapter had to possess a triplet offset mechanism.

*Third*, the adaptor should form the cavities to bind the amino acids, which should be directly associated with the anticodon, or be near the anticodon.

The extant tRNA or any other adaptor based on the double-helix would not meet the requirements since its only "measuring stick"—the diameter of the RNA duplex (2.3. nm)—is significantly larger than the length of the triplet (~ 1 nm). Even if the duplex diameter would match the triplet length, placing two negatively charged duplexes side by side with facing phosphates to each other would be rather problematic. In addition, double-helical adaptors do not have the codon length offset mechanism, and the amino acid attachment site is too far from the anticodon (76 Å).

Surprisingly, QD meets all the requirements.

*First*, the stretch of three G-tetrads perfectly matches the stretch of three base pairs. Additionally, QDs readily form higher-order assemblies through stacking interactions at both ends [31, 32] (Fig. 8).

*Second*, Z-looping can be achieved by as little as one and two nucleotides creating both meaningful and meaningless positions (spacers). The various combinations of these positions create the triplet offset mechanism. Figure 8 demonstrates schematics of all three possibilities: QD binding to the mRNA through (i) one base-pair with two spacers, (ii) two base-pairs with one spacer, and (iii) three base-pairing.

*Third*, as mentioned above, Z-shaped loops of QD are stretched over the quadruplex grooves without crossing the helical axes. As a result, three cavities between the grooves and the loops are formed (see Fig. 3), representing potential amino acid attachment sites. By entering the cavity, the amino acid can directly attach to the cognate anticodon through the sugar-phosphate backbone and the atomic groups of the bases not involved in codon-anticodon interaction, probably at the sugar edge [53]. Thus, QD loops can directly bind and associate two critical components of the translation—the codon and the amino acid.

## QD as a primitive translation apparatus

The extant translation is the multistep and highly sophisticated process fully governed by the ribosome and companion factors. Without the translational apparatus, most of the steps are just physically impossible. Surprisingly, all steps of QD-mediated translation are feasible without violating the continuity principle.

**Fig. 9:**
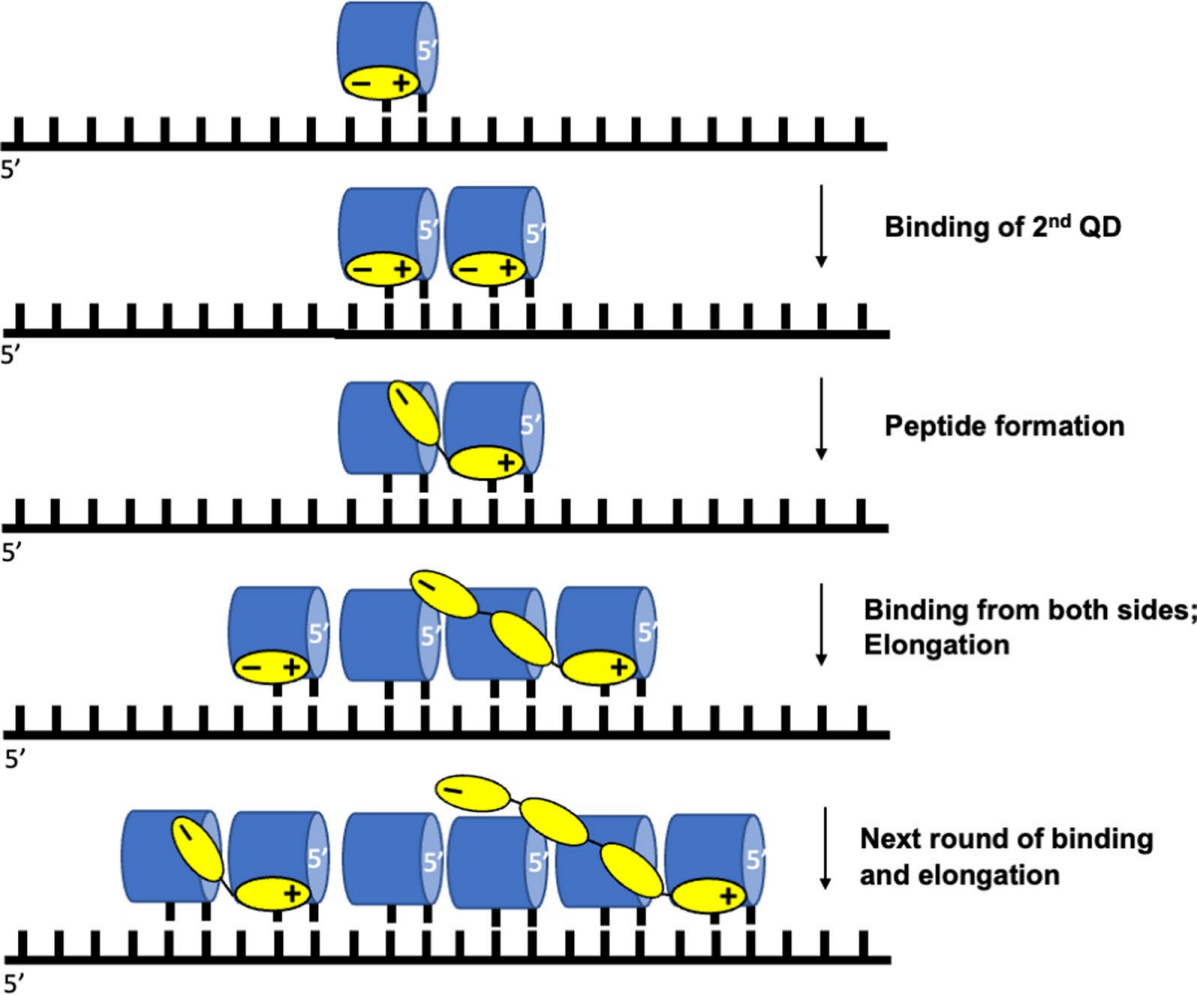
Pathway of QD-conducted translation. The QDs are represented as blue cylinders. The amino acids are presented as yellow ovals with N- and C-termini designed with plus and minus signs

**Fig. 10:**
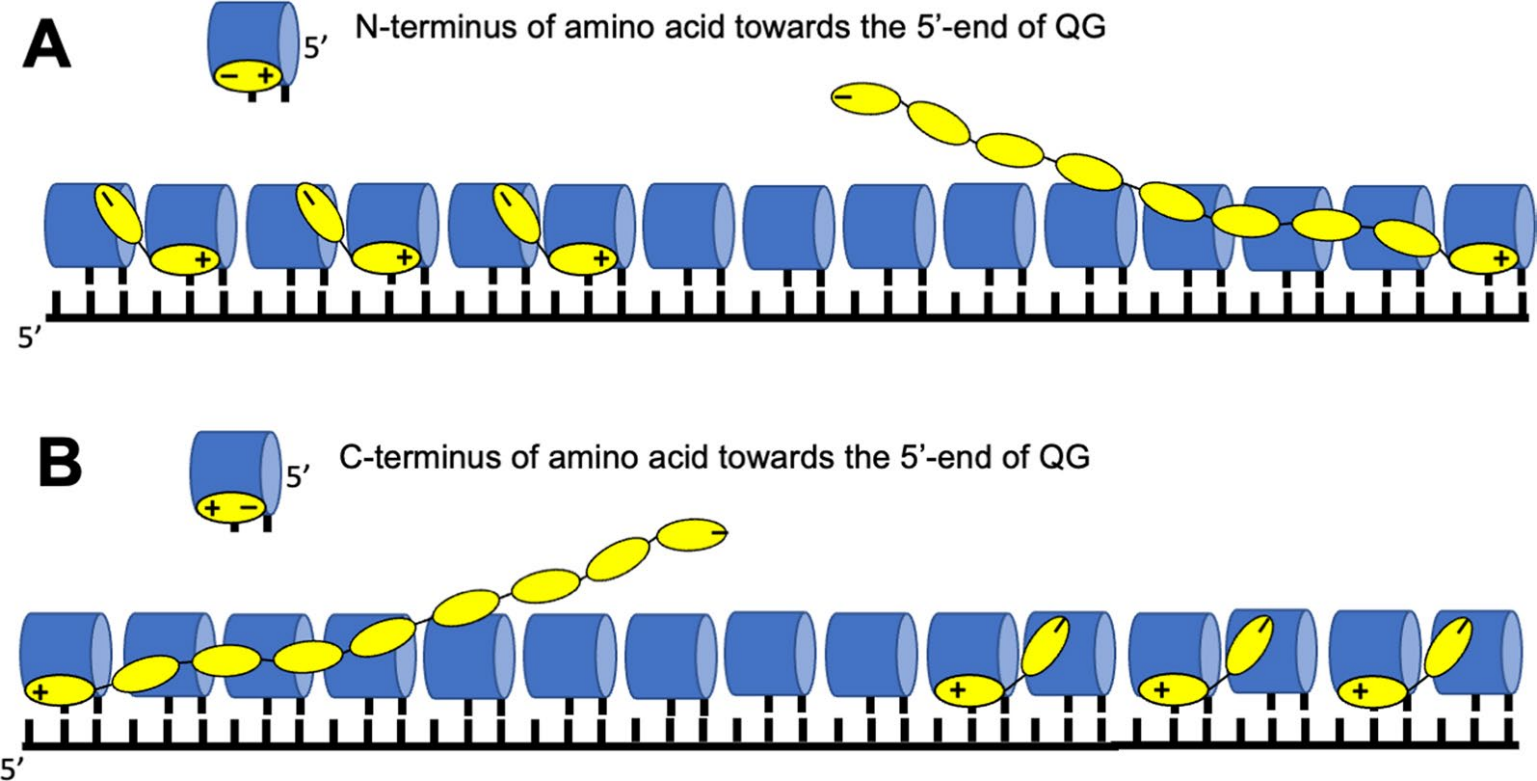
Translation using QDs with differently oriented amino acids. N-terminus of the amino acid is located towards the 5′-end (**A**) and towards the 3′-end (**B**) of the QD. The QDs are represented as blue cylinders. The amino acids are presented as yellow ovals with N- and C-termini designed with plus and minus signs

As mentioned above, the amino acid can bind to the cavities between the loops and quadruplex grooves. The positively charged amine group of an amino acid would interact with the negatively charged loop phosphates, while negatively charged carboxyl group would be exposed outwards due to the overall anionic nature of the QD. As a result, the QD-attached amino acids would have a similar orientation. Since each QD has more than one loop, two alternative charging ways should be discussed: (i) the amino acid attachment to the anticodon or the loop involved in codon binding; and (ii) amino acid attachment to a loop not involved in the codon binding. The latter version is more problematic since it requires QDs with at least two identical loops and a specific mechanism to align the anticodons with the amino acid attachment sites. However, the former version is free from these requirements and, therefore, is employed in the hypothetical translation scenario discussed below. This variant uses QDs with 2-nt loops and a carboxyl group of the amino acid oriented towards the 5'-end of the QD (Figs. 9 and 10A), for the opposite orientation, see Fig. 10B.

Binding of the first QD to mRNA (note, this is the first encounter with the complementarity principle), at any position (see Fig. 9), is followed by binding/stacking of the second QD at the 5'-end of the first QD. The binding at the 3'-end would not affect overall translation process and its direction since the orientation of the amino acids determines it. Specifically, upon peptide bond formation, the charges at the reactive moieties disappear; in the newly produced dipeptide, the first amino acid becomes anionic, and the second amino acid becomes cationic. As a result, the C-terminus dissociates from the cognate QD, while the N-terminus binds even stronger to its cognate QD (Fig. 9). Thus, the dipeptide stays with the incoming QD, similar to the extant system. Because of this mechanism, the binding of QDs at 3'-end of the discharged QDs can create only the dimers (Figs. 9 and 10). For simplicity, the dissociation of the discharged QD isn't shown. It could be facilitated by discharging QDs (amino acid dissociation) or by a temperature cycle.

The reaction describes in Fig. 9 represents one of the simplest ways to produce the peptides. Similar processes could be envisaged for QDs with 1-nt or 3-nt loops with non-covalently or covalently attached amino acids. The latter could proceed by binding amino acids at the closest loops to the 3'-end of QD (including the operational code, see Fig. 7) followed by amino acid condensation to the terminal ribose.

## Quadruplex world is not a prelude to RNA world

As mentioned above, both DNA and RNA QDs with single-nt loops fold into the all-parallel quadruplex shown in Fig. 3. The loop elongation doesn't affect the folding topology of the RNA QDs, while it introduces the structural polymorphism in DNA QDs. However, under the low water activity, DNA QDs with three-nt loops maintain their all-parallel topology [41]. Therefore, it is more likely that the quadruplex world was employing RNA sequences; however, DNA or hybrid (DNA/RNA) sequences can't be excluded.

The quadruplex world originates from the free guanines organizing G-tetrad crystalline structures, which is followed by polymerization and folding of the monomolecular quadruplexes, QDs (Fig. 2B). This process is solely based on the G-tetrads without employing the WC base-pairing rules and can be attributed to the quadruplex world. After forming the QDs, the evolution is ready to initiate the primitive translation process, shown in Fig. 9. The process includes an amino acid attachment to QDs and binding the charged QDs to the mRNA. The latter is achieved through WC base-pairing. Thus, the evolution adds to its tool-box base pairing rules at this stage. However, it is not capable to perform the replication yet. Therefore, the process described in Fig. 9 can be called the “quadruplex-protein world”. After introducing the replication and producing RNA duplexes, the quadruplex-peptide world would transform into the “RNA–protein world”. So, the quadruplex approach of the origin of life allows step-by-step transformations of free bases into the extant system: quadruplex world → quadruplex-protein world → RNA–protein world. Notably, the quadruplex-peptide world is the result of appending of the amino acids into the quadruplex world, not simultaneous appearing and co-evolution of the protein and RNA precursors suggested by the “peptide-RNA world” [54].

## Conclusion

The quadruplex world hypothesis can reconstruct origin of the translation apparatus as a sequence of reasonably realistic events starting from free G-monomers. According to this scenario, the life begins from the crystalline structures of G-monomers employing G-tetrads as a self-assembly element. The G-tetrads stack on each other with the same helical parameters as the quadruplexes formed by polyG molecules. This juxtaposes reactive moieties of the G-monomers and facilitates polymerization. So, the very first polynucleotide is synthesized from the G-monomers in a single-step reaction without any external help (e.g., clay minerals).

After reaching the necessary length, G_15_, the oligomer folds into the quadruplex domain or QD with strictly defined 3D architecture: three G-tetrads connected by three Z-shaped G-loops. The QDs form with extraordinarily favorable thermodynamics. As a result, QD formation is highly programmable, and the genotype–phenotype relationship in the quadruplex world is unambiguous.

The QD architecture has a multifaceted surface and unique structural properties that allow QD to play the role of an early adaptor and singlehandedly conduct a primitive translation: (i) the QD building pattern accepts only three G-tetrads (this should be the determining factor of the codon length); (ii) the end G-tetrads are cleared for stacking interaction with other QDs; (iii) the loop bases are oriented perpendicular to the main body of QD. As a result, QDs can simultaneously base-pair with the mRNA (through the loop base) and stack to each other (through the end G-tetrads) in parallel to mRNA; (iv) Z-loops can be formed by as short as single-nt loops. This property, in the combination of the triplet nature of QDs, allows to have a triplet offset mechanism of the codons and expand the genetic code without the codon length elongation; (v) Z-loops create cavities for the amino acid attachment, and upon QD alignment on mRNA can facilitate peptide bond formation. Evolution already has the primitive translation apparatus producing some oligopeptides, and the quadruplex world transforms into the quadruplex-protein world before introducing the replication (base-pairing is used only for QD binding to mRNA). Thus, the quadruplex world takes several necessary evolutionary steps and transforms into the quadruplex-protein world before introducing the replication process.

## Data Availability

All data and materials generated or analyzed during the study are included in the published article.

## Abbreviations

3D: Three-dimensional
QD: Quadruplex domain
G: Guanine
nt: Nucleotide
RCR: Rolling circle replication
PCR: Polymerase chain reaction
